# Acute liver failure in Still’s disease relapse during pregnancy: case report and discussion of a possible trigger role of DILI

**DOI:** 10.1186/s12876-021-01878-3

**Published:** 2021-08-06

**Authors:** Giuseppe Marrone, Francesco Galati, Marco Biolato, Christopher Oddy, Sara De Carolis, Angelo Zoli, Antonio Grieco

**Affiliations:** 1 Transplant Hepatology Unit - CEMAD Digestive Disease Center, Fondazione Policlinico Universitario “A. Gemelli” IRCCS, Università Cattolica del Sacro Cuore, Largo A. Gemelli 8, 00168 Rome, Italy; 2 FY2 Intensive Care Medicine, Epsom & St Helier University Hospitals NHS Trust, Epsom, UK; 3 Obstetrics and Obstetric Pathology Unit, Fondazione Policlinico Universitario “A. Gemelli” IRCCS, Università Cattolica del Sacro Cuore, Rome, Italy; 4 Osteo-articular Disease Unit, Fondazione Policlinico Universitario “A. Gemelli” IRCCS, Università Cattolica del Sacro Cuore, Rome, Italy

**Keywords:** Acute onset Still’s disease, Acute liver failure, Drug induced liver failure, ALF case report

## Abstract

**Background:**

Still's disease is a rare systemic inflammatory disease with frequent but generally mild liver involvement. The most common cause of acute liver failure in western countries is drug-induced liver injury, while it has rarely been reported in subjects suffering from Still’s disease.

**Case presentation:**

We report a case of a young woman presenting with SD reactivation in pregnancy and acute liver failure after delivery with a possible triggering role of drug induced liver injury.

**Conclusions:**

The prompt recognition of Still's disease reactivation allowed early introduction of steroid therapy and resolution of the clinical picture. We discuss potential factors precipitating ALF in this case, and implications for the diagnosis and management of such patients.

## Background

Acute Liver Failure (ALF) refers to a specific syndrome characterized by abnormal liver function tests, coagulopathy and altered level of consciousness due to hepatic encephalopathy (HE) in a patient with recent onset liver damage (< 26 weeks) [1]. ALF has numerous potential causes, among which the most frequent are: acute viral and alcoholic hepatitis, drug induced liver injury (DILI), cryptogenic liver failure, hepatic vascular disease (including Budd-Chiari syndrome), Wilson’s disease and pregnancy associated acute liver disease (PAALD) [2, 3]. ALF may also develop as a result of acute presentation of autoimmune liver disease, rarely in cases of Systemic-Onset Juvenile Idiopathic Arthritis (SOJIA) [4, 5].

SOJIA is a systemic inflammatory disorder characterized by fever and arthritis, accompanied by at least one of the following: rash, generalized lymphadenopathy, hepatomegaly ± splenomegaly, and serositis (Table 1). However, the classic features are not always present at disease onset [6]. Contemporary opinion considers SOJIA and Adult-Onset Still’s Disease (AOSD) as a disease continuum with different ages of onset (before or after 16 years of age), but with the same main characteristics and extremely similar gene-expression, clinical course, prognosis, and responsiveness to therapy [7–11].

**Table 1:** Yamaguchi criteria for diagnosis of adult-onset Still’s disease

Yamaguchi criteria	
Major criteria	
Fever of at least 39 °C for at least a week	√
Arthralgia or arthritis for at least 2 weeks	√
Non-pruritic salmon colored rash on trunk/extremities	√
Granulocytic leukocytosis (10,000/µL or greater)	√
Minor criteria	
Sore throat	
Lymphadenopathy	
Hepatomegaly or splenomegaly	
Abnormal liver function tests	√
Negative tests for RF and ANA	√

The diagnosis requires at least 5 features, two of which being major diagnostic criteria. **√** indicates the criteria met in the presented case

In some published series, a higher frequency of liver damage has been reported in the adult form [12].

We present a case of ALF during pregnancy in a young woman with previous history of SOJIA with a potential concomitant DILI which may have acted as a trigger for severe SD reactivation.

## Case presentation

A 20-year-old woman, with previous history of SOJIA, was referred to our Centre in January 2019 for management of ALF that developed following a preterm delivery. SOJIA was diagnosed when she was three years old after which she experienced several remissions and exacerbations—characterized by arthralgias, fevers and rashes—until the age of 14. Subsequently, she started anakinra therapy achieving stable remission of the disease, with treatment discontinuation at the age of 16. She had her first pregnancy at the age of 18, with term delivery and no obstetric complications or disease exacerbations.

During the fourth month of her second pregnancy, because of the development of widespread arthralgias and fevers (up to 40 °C), steroids were prescribed (prednisone 15 mg/day, later tapered to 7.5 mg/day). Following initiation of steroids there was resolution of her fevers but little improvement in her joint pain. This prompted self-administration of high dose acetaminophen (5–6 g/day) for at least 45 days.

At 22-weeks gestation, subcutaneous certolizumab pegol (twice monthly) was added with clinical improvement in her symptoms and reduction of acetaminophen dosage to 1–2 g/day. Blood tests revealed normal transaminases and bilirubin levels. INR value was not assessed at this point, but it was normal in the early gestation.

**Fig. 1:**
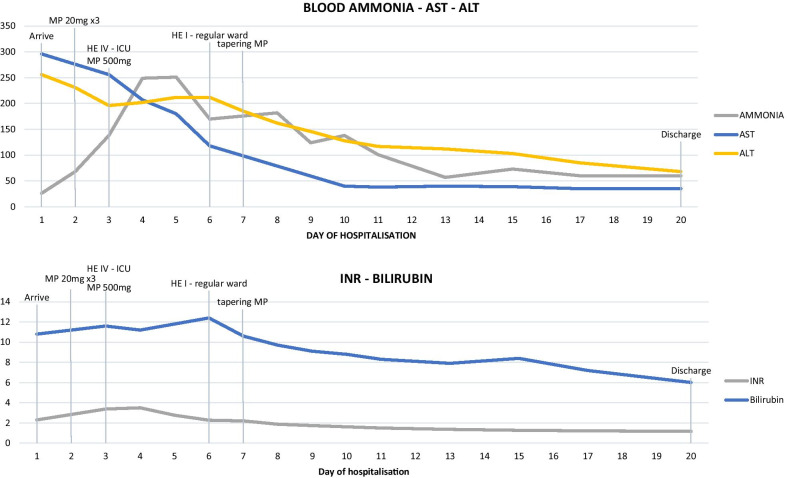
Trends of blood chemistry during hospital stay and in relation to relevant clinical events and treatments. Abbreviations: *MP* methylprednisolone, *HE* hepatic encephalopathy according to West Haven criteria, *HE I WE* Grade I Hepatic Encephalopathy according to West Haven criteria, *ICU* intensive care unit

At 28-weeks gestation, she was hospitalized because of development of jaundice. Blood tests revealed raised transaminases (AST 1499 IU/L, ALT to 1085 IU/L), hyperbilirubinemia (total bilirubin 7.3 mg/dl, unconjugated bilirubin 6.9 mg/dl) and coagulopathy (INR 1.68) (Fig. 1). In addition to prednisone, treatment with glutathione, ursodexoxycholic acid and N-acetylcysteine was started. There was improvement of transaminases (AST 555 IU/L. ALT 554 IU/L), slight reduction of bilirubin levels (5.8 mg/dl) but worsening of coagulopathy (INR 2.3, coagulative factor V 59%). Ammonia levels were still within the normal range (Fig. 1).

7 days after hospitalization, at 29 weeks gestation, she gave birth spontaneously by vaginal delivery to a male child weighing 1180 g. Soon after delivery she developed fever and a facial rash. At this point she was transferred to our hospital with the suspicion of an evolution towards ALF. On admission the patient was alert and febrile (> 39 °C), with no neurological impairment. A salmon coloured maculo-papular skin rash involving the trunk, back and neck was present. She complained about widespread arthralgias.

Laboratory work-up demonstrated reduction of transaminases (AST 170 IU/L, ALT 248 IU/L), worsening of hyperbilirubinemia (total bilirubin 15.2 mg/dl), stable coagulopathy (INR 2.3), mild hyperferritinemia (327 ng/ml) and increase in white blood cell count (21.5/mm^3^) with neutrophils 86.7% and absence of ANA, ASMA, AMA, anti-LKM, anti-dsDNA, and ANCA serum antibodies (Table 1). Abdominal CT was also normal. Within the first 48 h of admission at our centre the patient remained stable with no clinical signs of HE, falling transaminases and stable bilirubin levels.

Given her history of SOJIA, and both clinical and laboratory findings suggesting a SD reactivation (Table 1), methylprednisolone therapy was initiated at 20 mg TID with remission of fever but persistence of her rash. After three days, laboratory tests showed an increasing INR (2.9). Coagulation factor V was 44.5%, bilirubin levels were stable (15 mg/dl) and transaminases were decreasing (ALT 190 IU/L), but venous ammonia levels were increasing (190 ug/dl) (Fig. 1). A progressive worsening in neurological function occurred with psychomotor agitation, confusion and inappropriate speech attributed to a grade III hepatic encephalopathy (HE) [13]. A cerebral CT scan was performed and was broadly unremarkable. EEG showed signs of global encephalopathy.

The daily dosage of methylprednisolone was further increased (500 mg QD), antibiotic therapy with meropenem was started and the patient was moved to ICU. Her neurological condition worsened to grade IV HE, without the need for mechanical ventilation. During her ICU stay the patient did not meet the King's College Criteria for emergency liver transplant [13, 14]. High dose corticosteroid therapy was continued for three days and subsequently tapered following progressive spontaneous recovery of her neurological condition and laboratory data. Antibiotic therapy was stopped after neurological improvement. The patient was moved to a regular ward after six days.

**Fig. 2:**
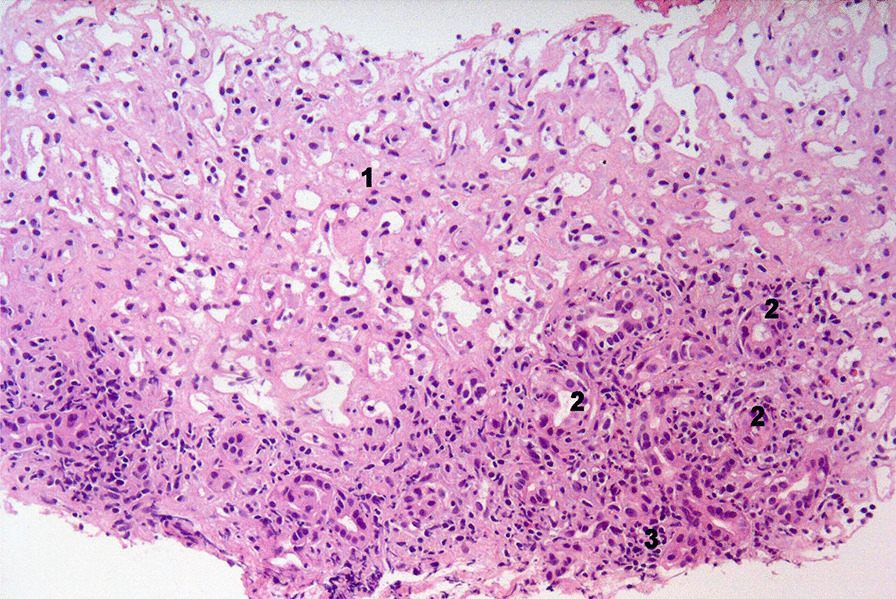
×20 Liver histology image. Liver sample was obtained at the normalization of coagulation parameters. 1—confluent hepatocyte necrosis; 2—cholestasis with ductular reaction; 3—eosinophilic granulocyte infiltrate. Masson's trichrome staining documented the presence of mild to moderate fibrosis. Immunohistochemistry showed marked activation of Kupffer cells but without plasma cell infiltrate (image not shown)

After resolution of coagulation parameters, a percutaneous liver biopsy was performed. Liver histology revealed severe necro-inflammatory activity with large areas of confluent hepatocytic necrosis and cholestatic/cholangitic aspects and mild fibrosis. A marked Kupffer cell activation was also observed without plasma cell infiltrate. An eosinophilic portal infiltrate was also present. The fibrosis was scored as mild-to moderate. Perls staining for hemosiderin was negative (Fig. 2). In the following days, the patient progressively improved and was discharged on the 20th day (Fig. 1). The patient started an outpatient follow-up program on low dose oral steroids. A transient relapse with a contemporaneous increase in transaminases and back rash was effectively treated with increase in steroid dosage. The patient continued low dose steroid therapy with clinical benefit and was subsequently lost to follow-up after six months.

## Discussion and conclusions

A peculiar case of ALF with associated SD relapse after delivery, with a possible component of DILI, is reported.

At presentation, PAALD (hyperemesis gravidarum, intrahepatic cholestasis of pregnancy, HELLP syndrome) was considered but was ruled out during the initial clinical assessment. Acute fatty liver of pregnancy (AFLP) was later excluded due to the complete absence of steatosis at liver biopsy.

The relationship between SD and pregnancy has been insufficiently examined, and much of the data is conflicting. Recently, a review by De Carolis et al. [15] clarified that an increase in relapses can occur in the first and second trimester of pregnancy with an increased risk of obstetric complications, such as preterm delivery, intrauterine growth restriction and neonatal hemophagocytic lymphohistyocytosis, while other reports in women with a previous diagnosis of SOJIA reported a relatively stable disease activity during pregnancy with postpartum exacerbation [16].

The reported history of long-term (approximately 45 days) consumption of high daily dose of acetaminophen (4–6 g/day) during pregnancy was considered among the cause of ALF. Most of the acetaminophen overdoses in pregnancy described in the literature are of single episodes of consumption, it is quite rare to find a staggered overdose [17]. Single episode acetaminophen overdose occurs when a single supratherapeutic dose of paracetamol (> 4 g) is taken [18]. A staggered overdose, as in our case, is the ingestion of two or more supratherapeutic doses over a time interval longer than 8 h [19, 20]. In this case the Roussel Uclaf Causality Assessment Method (RUCAM) calculated for acetaminophen toxicity gave a score of 5, classifying DILI as a “possible” aetiology. It is, however, worth noting that symptoms appeared when drug dosage had already been reduced. Moreover, a few weeks before the development of acute liver injury (ALI) the patient was started certolizumab pegol, with reduction of daily paracetamol amount. Certolizumab, therefore, may also be implicated.

Certolizumab is a monoclonal anti-TNFα antibody. This class of drugs is known to cause DILI or drug induced autoimmune hepatitis (DIAIH) [21–24]. In our case DIAIH is unlikely based on liver histology and the negativity of autoantibodies [25]. Anti-TNFα-induced ALF is a rare occurrence and has been reported for infliximab, and less frequently, for adalimumab [22–29] while only one case of DILI by certolizumab has been described in the literature, but no case of ALF [30]. The RUCAM calculated for certolizumab is 6, placing a diagnosis of DILI by certolizumab as "probable". Although certolizumab may have contributed to liver injury, the presence of symptoms of SD reactivation at the time of clinical worsening and improvement after high dosage steroids treatment make us consider SD reactivation as the principal cause of ALF.

A further hypothesis we considered is whether DILI may have caused the reactivation of SD. Recent evidence has highlighted the role of the immune system in idiosyncratic DILI [31–33] and it would therefore be plausible to speculate a role of DILI in SD reactivation, even if this is not described in the literature so far. Considering the two involved drugs, certolizumab was certainly administered at non-toxic doses and the temporal latency of liver injury may be possibly compatible with an idiosyncratic DILI. However, although expected, no drug-induced hepatic autoimmune manifestations were observed in liver histology. In our case, pregnancy can certainly be considered the most important triggering factor of SD reactivation, which occurred prior to the administration of the two drugs. Onset and reactivation of SD during pregnancy have been described in various reports, with different courses and different rates of obstetric complications [15]. In our patient, after long a period of clinical remission without the need of pharmacological treatment, a reactivation of the disease occurred during the second trimester. Data regarding SD and pregnancy are conflicting in the literature and it is difficult to compare the course of the disease in subjects in clinical remission during medical treatment and untreated subjects, as in our patient [16, 34, 35]. Although pregnancy may have caused the initial reactivation of the disease in the 2nd trimester, other factors came into play determining the clinical picture of ALF. Considering the time course of the disease, we must note that ALI was improving at the time of SD exacerbation during delivery. It is well known that transaminases reduction during ALI is not always a good news, as it may correspond to an exhaustion of the hepatic functional mass [36], but, in our case, the rapid subsequent functional recovery runs counter to such an interpretation. We can therefore speculate that DILI caused ALI and, in a clinical context of impaired immunity, DILI itself exacerbated SD which acted as a “second hit” on liver function, precipitating the picture towards a frank ALF. Moreover, the peculiar immunological peri-partum condition could have influenced the clinical course of the disease. SD exacerbation occurred during delivery and progression to ALF in the following days, when the pregnancy-related immunological deviation stopped, and post-partum immune rebound was starting. Our patient may therefore have faced a multifactorial immunological storm condition, causing an extremely severe SD reactivation. In most of the reported cases of ALF in SD, a macrophage activation syndrome (MAS) was observed [37]. In our case MAS criteria were not satisfied [38] but the synergic combination of still-related liver damage superimposed on DILI resulted in ALF.

Skin lesions are also described during DILI with immune-allergic mechanism, but in our case, the absence of eosinophilia or other allergic manifestations, such as itching, angioedema or wheeze, and the typical aspect of the rash (salmon coloured, maculo-papular, non-pruritic), consistent with SD, makes the rheumatological nature of the lesions more likely.

Hepatic involvement in the course of SD is frequent and it is among Yamaguchi's minor criteria for the diagnosis of SD [39], but usually presents as only a mild increase in transaminases [40]. The occurrence of ALF is a very rare occurrence and few cases have been reported in the literature [41–46], with 7 of them (46.7%) [41–47] requiring liver transplantation (LT).

The value of liver biopsy in the diagnosis of SD remains a topic for debate [48] as highly variable histological features have been described. The most common histological finding is Kupffer cell hyperplasia accompanied by periportal inflammatory infiltrates, but the presence of portal fibrosis, hepatocytic necrosis and peliosis has also been described [5, 49, 50]. Considering the results of liver biopsy in our patient, whilst a non-specific pattern was found, it was considered not incompatible with a diagnosis of a SD-induced acute liver damage. The observed histological picture could be also attributable to DILI which, as discussed above, may have played a role in the reactivation and worsening of SD-induced liver damage. The described histological picture is not exclusive of either SD or DILI therefore, this data alone does not allow to attribute the observed damage to one of the two conditions. The execution of liver biopsy instead allowed to rule out some conditions which come in differential diagnosis in the described case, as AFLP and Autoimmune Hepatitis (AIH). Notably, liver biopsy was performed several days after the critical phase of the disease, following administration of high dose steroids, and thus we observed a histological picture that might have been influenced by this treatment.

Reactivation of SD during pregnancy is a crucial clinical condition that should always be kept in mind in the presence of a history of known SOJIA. Liver injury is frequent during SD reactivation and it can occasionally evolve to ALF. The use of drugs with potential hepatotoxic effect in the treatment of SD reactivation can make it difficult to determine the cause of liver injury. In the case of SD-induced liver injury, prompt diagnosis and administration of high dose steroids is essential to alter the clinical course of the disease, avoiding the need of an emergency LT.

## Data Availability

Data sharing is not applicable to this article as no datasets were generated or analysed during the current study.

## Abbreviations

AFLP: Acute fatty liver of pregnancy
AIH: Autoimmune hepatitis
ALF: Acute liver failure
ALI: Acute liver injury
AOSD: Adult-onset Still’s disease
DIAIH: Drug induced autoimmune hepatitis
DILI: Drug induced liver injury
HE: Hepatic encephalopathy
LT: Liver transplantation
MAS: Macrophage activation syndrome
PAALD: Pregnancy associated acute liver disease
RUCAM: Roussel Uclaf Causality Assessment Method
SOJIA: Systemic-Onset Juvenile Idiopathic Arthritis

## References

[1] Association E (2017). EASL Clinical Practical Guidelines on the management of acute (fulminant) liver failure. J Hepatol.

[2] Bernal W, Auzinger G, Dhawan A, Wendon J (2010). Acute liver failure. Lancet.

[3] Chalasani N, Fontana RJ, Bonkovsky HL, Watkins PB, Davern T, Serrano J, Yang H, Rochon J (2008). Causes, clinical features, and outcomes from a prospective study of drug-induced liver injury in the United States. Gastroenterology.

[4] Ferro F, Cioffi E, Elefante E (2014). AB0925 liver involvement in adult onset Still’s disease: retrospective analysis of 18 cases. Ann Rheum Dis.

[5] Lim KBL, Schiano TD (2011). Still disease and the liver-an underappreciated association. Gastroenterol Hepatol N Y.

[6] Lee JJY, Schneider R (2018). Systemic Juvenile Idiopathic Arthritis. Pediatr Clin N Am.

[7] Cabane J, Michon A, Ziza JM, Bourgeois P, Blétry O, Godeau P, Kahn MF (1990). Comparison of long term evolution of adult onset and juvenile onset Still’s disease, both followed up for more than 10 years. Ann Rheum Dis.

[8] Jamilloux Y, Gerfaud-Valentin M, Martinon F, Belot A, Henry T, Sève P (2014). Pathogenesis of adult-onset Still’s disease: new insights from the juvenile counterpart. Immunol Res.

[9] Nirmala N, Brachat A, Feist E, Blank N, Specker C, Witt M, Zernicke J, Martini A, Junge G (2015). Gene-expression analysis of adult-onset Still’s disease and systemic juvenile idiopathic arthritis is consistent with a continuum of a single disease entity. Pediatr Rheumatol.

[10] Feist E, Mitrovic S, Fautrel B (2018). Mechanisms, biomarkers and targets for adult-onset Still’s disease. Nat Rev Rheumatol.

[11] Luthi F, Zufferey P, Hofer MF, So AK (2002). “Adolescent-onset Still’s disease”: characteristics and outcome in comparison with adult-onset Still’s disease. Clin Exp Rheumatol.

[12] Nuran SP, Mukaddes T, İsmail K (2006). A multicenter study of patients with adult-onset Still’s disease compared with systemic juvenile idiopathic arthritis. Clin Rheumatol.

[13] O’Grady J, Alexander G, Hayllar K, Williams R (1989). Early indicators of prognosis in fulminant hepatic failure. Gastroenterology.

[14] McPhail MJW, Wendon JA, Bernal W (2010). Meta-analysis of performance of Kings’s College Hospital Criteria in prediction of outcome in non-paracetamol-induced acute liver failure. J Hepatol.

[15] De Carolis S, Cianci F, Del Sordo G, Garofalo S, Garufi C, Lanzone A, Zoli A, Gremese E (2019). Adult onset Still’s disease and pregnancy. Autoimmun Rev.

[16] Ursin K, Lydersen S, Skomsvoll JF, Wallenius M (2018). Disease activity of juvenile idiopathic arthritis during and after pregnancy: a prospective multicenter study. J Rheumatol.

[17] Thornton SL, Minns AB (2012). Unintentional chronic acetaminophen poisoning during pregnancy resulting in liver transplantation. J Med Toxicol.

[18] Zimmerman HJ, Maddrey WC (1995). Acetaminophen (paracetamol) hepatotoxicity with regular intake of alcohol: analysis of instances of therapeutic misadventure. Hepatology.

[19] Craig DGN, Bates CM, Davidson JS, Martin KG, Hayes PC, Simpson KJ (2012). Staggered overdose pattern and delay to hospital presentation are associated with adverse outcomes following paracetamol-induced hepatotoxicity. Br J Clin Pharmacol.

[20] Larson AM, Polson J, Fontana RJ (2005). Acetaminophen-induced acute liver failure: results of a United States multicenter, prospective study. Hepatology.

[21] Andrade RJ, Aithal GP, Björnsson ES, Kaplowitz N, Kullak-Ublick GA, Larrey D, Karlsen TH (2019). EASL Clinical Practice Guidelines: drug-induced liver injury. J Hepatol.

[22] Ghabril M, Bonkovsky HL, Kum C, Davern T, Hayashi PH, Kleiner DE, Serrano J, Rochon J, Fontana RJ, Bonacini M (2013). Liver injury from tumor necrosis factor-α antagonists: analysis of thirty-four cases. Clin Gastroenterol Hepatol.

[23] Efe C, Purnak T, Ozaslan E, Wahlin S (2010). Drug-induced autoimmune hepatitis caused by anti-tumor necrosis factor α agents. Hepatology.

[24] Lopetuso LR, Mocci G, Marzo M, D’aversa F, Rapaccini GL, Guidi L, Armuzzi A, Gasbarrini A, Papa A, (2018). Harmful effects and potential benefits of anti-tumor necrosis factor (TNF)-α on the liver. Int J Mol Sci.

[25] Mack CL, Adams D, Assis DN, Kerkar N, Manns MP, Mayo MJ, Vierling JM, Alsawas M, Murad MH, Czaja AJ (2020). Diagnosis and management of autoimmune hepatitis in adults and children: 2019 practice guidance and guidelines from the American Association for the study of liver diseases. Hepatology.

[26] Tobon GJ, Cañas C, Jaller JJ, Restrepo JC, Anaya JM (2007). Serious liver disease induced by infliximab. Clin Rheumatol.

[27] Hagel S, Bruns T, Theis B, Herrmann A, Stallmach A (2011). Subacute liver failure induced by adalimumab. Int J Clin Pharmacol Ther.

[28] Kinnunen U, Färkkilä M, Mäkisalo H (2012). A case report: Ulcerative colitis, treatment with an antibody against tumor necrosis factor (infliximab), and subsequent liver necrosis. J Crohn’s Colitis.

[29] Kok B, Lester ELW, Lee WM, Hanje AJ, Stravitz RT, Girgis S, Patel V, Peck JR, Esber C, Karvellas CJ (2018). Acute liver failure from tumor necrosis factor-α antagonists: report of four cases and literature review. Dig Dis Sci.

[30] Ling C, Gavin M, Hanson J, McCarthy DM (2018). Progressive epigastric pain with abnormal liver tests in a patient with Crohn’s disease: don’t DILI Dally. Dig Dis Sci.

[31] Fontana RJ (2014). Pathogenesis of idiosyncratic drug-induced liver injury and clinical perspectives. Gastroenterology.

[32] Kullak-Ublick GA, Andrade RJ, Merz M, End P, Benesic A, Gerbes AL, Aithal GP (2017). Drug-induced liver injury: recent advances in diagnosis and risk assessment. Gut.

[33] Yokoi T, Oda S (2021). Models of idiosyncratic drug-induced liver injury. Annu Rev Pharmacol Toxicol.

[34] Gerfaud-Valentin M, Hot A, Huissoud C, Durieu I, Broussolle C, Seve P (2014). Adult-onset Still’s disease and pregnancy: about ten cases and review of the literature. Rheumatol Int.

[35] Remaeus K, Johansson K, Askling J, Stephansson O (2017). Juvenile onset arthritis and pregnancy outcome: a population-based cohort study. Ann Rheum Dis.

[36] EASL (2017). EASL Clinical Practical Guidelines on the management of acute (fulminant) liver failure. J Hepatol.

[37] Ravelli A, Grom AA, Behrens EM, Cron RQ (2012). Macrophage activation syndrome as part of systemic juvenile idiopathic arthritis: diagnosis, genetics, pathophysiology and treatment. Genes Immun.

[38] Ravelli A, Minoia F, Davì S (2016). 2016 Classification criteria for macrophage activation syndrome complicating systemic juvenile idiopathic arthritis: a European league against Rheumatism/American college of Rheumatology/Paediatric rheumatology international trials organisation collaborat. Ann Rheum Dis.

[39] Yamaguchi M, Ohta A, Tsunematsu T, Kasukawa R, Mizushima Y, Kashiwagi H, Kashiwazaki S, Tanimoto K, Matsumoto Y, Ota T (1992). Preliminary criteria for classification of adult Still’s disease. J Rheumatol.

[40] Zhu G, Liu G, Liu Y, Xie Q, Shi G (2009). Liver abnormalities in adult onset still’s disease: a retrospective study of 77 chinese patients. J Clin Rheumatol.

[41] Terán Á, Casafont F, Fábrega E, Martínez-Taboada VM, Rodríguez-Valverde V, Pons-Romero F (2009). Enfermedad de Still del adulto con desarrollo de insuficiencia hepática que precisa trasplante hepático. Gastroenterol Hepatol.

[42] Dino O, Provenzano G, Giannuoli G, Sciarrino E, Pouyet M, Pagliaro L (1996). Fulminant hepatic failure in adult onset Still’s disease. J Rheumatol.

[43] Yamanaka J, Saito S, Kuroda N, Hirano T, Fujimoto J (2003). Successful living related liver transplantation for adult still’s disease [1]. J Gastroenterol Hepatol.

[44] Liese J, Schreckenbach T, Wahle M, Welker MW, Ulrich F, Bechstein WO, Moench C (2012). Seltene Ursache eines akuten Leberversagens. Chirurg.

[45] Mcfarlane M, Harth M, Wall WJ (1997). Liver transplant in adult Still’s disease. J Rheumatol.

[46] Taccone FS, Lucidi V, Donckier V, Bourgeois N, Decaux G, Vandergheynst F (2008). Fulminant hepatitis requiring MARS and liver transplantation in a patient with Still’s disease. Eur J Intern Med.

[47] Ogata A, Kitano M, Yamanaka J, Yamasaki T, Hashimoto N, Iwasaki T, Hamano T, Fujimoto J, Kakishita E (2003). Interleukin 18 and hepatocyte growth factor in fulminant hepatic failure of adult onset Still’s disease. J Rheumatol.

[48] Andres E (2001). Liver biopsy is not useful in the diagnosis of adult Still’s disease. Qjm.

[49] Valluru N, Tammana VS, Windham M, Mekonen E, Begum R, Sanderson A (2014). Rare manifestation of a rare disease, acute liver failure in adult onset Still’s disease: dramatic response to methylprednisolone pulse therapy—a case report and review. Case Rep Med.

[50] Kim HA, Kwon JE, Yim H, Suh CH, Jung JY, Han JH (2015). The pathologic findings of skin, lymph node, liver, and bone marrow in patients with adult-onset still disease. Medicine.

