# Correction to: Acute liver failure in Still’s disease relapse during pregnancy: case report and discussion of a possible trigger role of DILI

**DOI:** 10.1186/s12876-021-01933-z

**Published:** 2021-10-01

**Authors:** Giuseppe Marrone, Francesco Galati, Marco Biolato, Christopher Oddy, Sara De Carolis, Angelo Zoli, Antonio Grieco

**Affiliations:** 1 Transplant Hepatology Unit - CEMAD Digestive Disease Center, Fondazione Policlinico Universitario “A. Gemelli” IRCCS, Università Cattolica del Sacro Cuore, Largo A. Gemelli 8, 00168 Rome, Italy; 2 FY2 Intensive Care Medicine, Epsom and St Helier University Hospitals NHS Trust, Epsom, UK; 3 Obstetrics and Obstetric Pathology Unit, Fondazione Policlinico Universitario “A. Gemelli” IRCCS, Università Cattolica del Sacro Cuore, Rome, Italy; 4 Osteo-articular Disease Unit, Fondazione Policlinico Universitario “A. Gemelli” IRCCS, Università Cattolica del Sacro Cuore, Rome, Italy

## Correction to: BMC Gastroenterol (2021) 21:317 10.1186/s12876-021-01878-3

After publication of this article [1], the authors noticed that the name of Christopher Oddy was incorrectly written as Cristopher Oddy.

The original article [1] has been updated.
